# Reversions to consensus are positively selected in HIV-1 and bias substitution rate estimates

**DOI:** 10.1093/ve/veac118

**Published:** 2022-12-15

**Authors:** Valentin Druelle, Richard A Neher

**Affiliations:** Biozentrum University of Basel, Spitalstrasse 41, Basel 4056, Switzerland; Swiss Institute of Bioinformatics, Spitalstrasse 41, Basel 4056, Switzerland; Biozentrum University of Basel, Spitalstrasse 41, Basel 4056, Switzerland; Swiss Institute of Bioinformatics, Spitalstrasse 41, Basel 4056, Switzerland

## Abstract

Human immunodeficiency virus 1 (HIV-1) is a rapidly evolving virus able to evade host immunity through rapid adaptation during chronic infection. The HIV-1 group M has diversified since its zoonosis into several subtypes at a rate of the order of 10^−3^ changes per site per year. This rate varies between different parts of the genome, and its inference is sensitive to the timescale and diversity spanned by the sequence data used. Higher rates are estimated on short timescales and particularly for within-host evolution, while rate estimates spanning decades or the entire HIV-1 pandemic tend to be lower. The underlying causes of this difference are not well understood. We investigate here the role of rapid reversions toward a preferred evolutionary sequence state on multiple timescales. We show that within-host reversion mutations are under positive selection and contribute substantially to sequence turnover, especially at conserved sites. We then use the rates of reversions and non-reversions estimated from longitudinal within-host data to parameterize a phylogenetic sequence evolution model. Sequence simulation of this model on HIV-1 phylogenies reproduces diversity and apparent evolutionary rates of HIV-1 in *gag* and *pol*, suggesting that a tendency to rapidly revert to a consensus-like state can explain much of the time dependence of evolutionary rate estimates in HIV-1.

## 1. Introduction

RNA viruses have low-fidelity polymerases, resulting in rapidly diversifying virus populations, which, in turn, facilitate the adaptation to changing environments. The human immunodeficiency virus 1 (HIV-1) is a prime example of such a rapidly evolving virus. The life-long infections it causes are characterized by a large viral population that accumulates diversity at a high rate to constantly evade host immunity (Coffin and Swanstrom 2013). This continuous evolution has led to a diverse viral population on the pandemic scale that is categorized into several viral subtypes (Brian Foley 2018; Li et al. 2015). Different lineages have accumulated diversity at a rate of about one substitution in 1,000 sites per year since its jump to human hosts at the turn of the 20th century (McCutchan 2006; Sharp, Hahn 2011; Korber et al. 2000).

Quantifying the rate of viral evolution, however, is surprisingly difficult and different approaches yield different answers. Most importantly, the timescale across which sequences are compared strongly affects the estimates, sometimes by orders of magnitude: the longer the timescale, the lower the estimate (Aiewsakun and Katzourakis 2016; Hanada et al. 2004; Worobey et al. 2010; Gilbert and Feschotte 2010; Ghafari et al. 2021). These discrepancies suggest that we lack a good understanding of how microevolutionary within-host (WH) processes—on the scales of days, months, and years—give rise to the diversity observed on longer timescales across hosts. In the case of chronic infections such as HIV-1, these microevolutionary processes are driven by selection to evade the host immune selection and mutations that reduce recognition can spread even if they reduce replication fitness. The pattern of immune selection changes at each transmission events and previously adaptive changes can become deleterious in the new host and sometimes revert (Leslie et al. 2004).

HIV-1 is an ideal system to study these effects in detail as the rate discrepancies among the WH, pandemic, and broader scales are well documented (Alizon and Fraser 2013; Worobey et al. 2010), the pandemic is well sampled, and high-resolution WH data exist. The evolutionary rate estimated on the pandemic scale is around two to five times lower than the one observed on the WH scale (Alizon and Fraser 2013). Several hypotheses have been put forward to explain this phenomenon. Two of the main hypotheses are the preferential transmission of ancestral HIV-1 variants, i.e. the ‘store and retrieve’ hypothesis (Lythgoe and Fraser 2012), and rapid reversion toward an ancestral-like state, i.e. the ‘adapt and revert’ hypothesis (Redd et al. 2012; Zanini et al. 2015; Leslie et al. 2004; Boutwell et al. 2010; Herbeck et al. 2006; Illingworth et al. 2020). The relative importance of these and possibly other processes for the discrepancy of rate estimates is not well understood (Raghwani et al. 2018).

We use WH longitudinal deep-sequencing data to explore how the rapid evolutionary processes within hosts can give rise to apparently lower rates on longer timescales. These results suggest that the ‘adapt and revert’ mechanism can explain most of the rate mismatch observed at different timescales of the HIV-1 pandemic. We, firstly, show that HIV-1 sequence evolution shows strong signs of site saturation while distance relative to the root of the tree increases much more slowly than expected based on the rate of evolution. Similar signatures are observed in longitudinal WH data, suggesting that this saturation is independent of whether evolution is quantified along transmission chains or within hosts. Secondly, we investigate the cause of this saturation and find that WH reversion toward the HIV-1 consensus is more common than expected and that such reversions are positively selected. Lastly, we use simulations of evolution to quantify the impact of rapid reversions on rate estimates for timescales of decades or more. These simulations show that the degree of reversion observed within hosts can explain the phylogenetic patterns observed in the pandemic. More generally, our results highlight the evolutionary bias of viruses toward a state of high intrinsic fitness in a changing environment.

## 2. Results

We use (1) a set of sequences representative of the HIV-1 pandemic spanning multiple decades and (2) a longitudinal data set following the evolution of the virus within individual hosts to investigate patterns of evolution on multiple timescales. The former between-host (BH) data set contains 1,000 HIV-1 group M sequences from the Los Alamos National Laboratory (LANL) HIV database (Foley et al. 2013). This subsampling was performed to have the same number of sequences for each year to avoid sampling biases (except for early years, where fewer sequences are available) but otherwise randomly picked from the full data set. The phylogenetic tree was inferred using an IQ-TREE GTR+F+R10 model (Tavaré and others 1986; Yang 1995; Minh et al. 2020), which was found to be the best model according to the IQ-TREE ModelFinder and allows for rate variation (Kalyaanamoorthy et al. 2017). For more details on the phylogenetic analysis and the estimates of rates, see Sections 4.2 and 4.3.

Our WH analysis is based on the HIVEVO data set (Zanini et al. 2015), a whole-genome deep sequencing of HIV-1 populations in eleven patients during a 4–16-year follow-up without treatment. Between six and twelve samples are available per patient, which typically cover 5–7 years of infection. Sequencing depth and template input of all samples in this data set have been assessed and most samples allow a confident calling of frequencies of minor variation down to a few per cent (Zanini et al. 2016). See Section 4.1 for details.

We analyze the evolution of the *env*, *pol*, and *gag* genes of HIV-1 Section 2.1 to 2.4. They code for surface proteins, viral enzymes, and capsid proteins, respectively (Freed 2001). When combined, they cover approximately 80 per cent of the genome. We focus on the *pol* region in the main text and present analogous results for the *env* and *gag* regions in the Supplementary Materials.

### 2.1 Saturation and reversion effects are comparable between and within hosts

**Figure 1.**
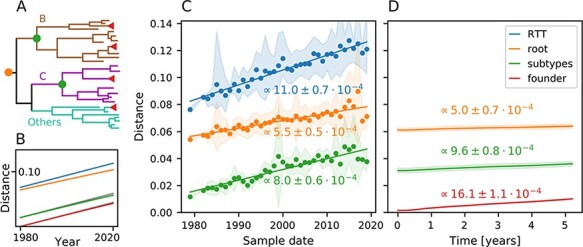
Divergence over time in the *pol* gene. (A) Sketch of the HIV-1 group M phylogenetic tree and its subtypes. Dots correspond to the position of the references used to compute distances in the other panels. WH evolution is indicated by red triangles. (B) Expected Hamming distances under a Jukes–Cantor (Jukes et al., 1969) evolution model with rate variation (gamma distributed, Parameter 2). Different curves show expected distance to the root of the tree (orange), subtype root (green), and WH founder (red). The blue and gray indicate linear growth of distance without saturations with a rate equal to the estimate from the root-to-tip (RTT) distance in Panel C. As expected, saturation effects are small since distances are around 10 per cent and multiple hits are rare. (C) Average Hamming distance from the root of the HIV-1 group M tree (orange), from the respective subtype (green, see dots in Panel A), or RTT distance in a phylogeny as a function of time. Each data point is the average of sequences from one year, lines are linear fits, and the shaded area indicates the 10–90 per cent range. (D) The WH divergence over time relative to the putative founder genotype, the HIV-1 group M root, and the subtype consensuses, averaged over all patients in the HIVEVO data set. Divergence is computed according to Equations (1) and (2). Standard estimates for the evolution rates BH and WH are the slopes of the RTT distance (blue) and divergence from founder sequence (red). There is an approximately 50 per cent difference between the evolution rates estimated while sequence distance is only a couple per cent. Comparing to the expectation (B), we can see that significant saturation of comparable magnitude can be seen on both BH (C) and WH (D) scales. Results for regions *env* and *gag* are shown in Supplementary Figs. S1 and S2.

The ‘adapt-and-revert’ mechanism to explain the rate mismatch within and between hosts assumes that reversions during WH evolution ‘shadow’ previous changes, resulting in very low rate estimates. The ‘store-and-retrieve’ mechanism postulates that many WH changes are not transmitted and thus irrelevant for the evolution on longer timescales (Lythgoe and Fraser 2012). To look for such discrepancies between WH and BH evolutionary patterns, we compared the rates at which sequences diverge away from the root of the HIV-1 tree or their subtypes at the BH and WH scales, see Fig. 1A.

The rate at which divergence between sequences increases decreases with distance as more and more sites are hit multiple times by mutations (Felsenstein, 2004). For very similar sequences multiple hits are negligible and divergence increases linearly in time with a slope given by the evolutionary rate. If all sites evolve at the same speed, such saturation effects are only important once distances between sequences are large (the size of correction is proportional to the distance squared and thus substantial if distances are 0.25 or larger). However, if different sites evolve at drastically different rates, or reversions to a preferred state are common, such saturation effects set in much earlier and can lead to significant deviations even when sequences are still very similar (Puller et al. 2020; Ghafari et al. 2021). The ‘adapt-and-revert’ mechanism thus posits strong saturation effects of similar magnitude both within and between hosts when compared to distant references such as the root of the HIV-1 M tree.

Sequences in the HIV-1 pandemic differ from each other at about 10–20 per cent of sites and we would naively expect that saturation effects are small unless rate variation is very strong or reversion is a substantial contribution to evolution. Figure 1B explores the observable consequences of such saturation on the scale of the HIV pandemic for a simple substitution model with gamma-distributed rate variation. The panel shows the evolutionary distance to the root of the tree corrected for saturation effects in blue. The latter is simply the evolutionary rate times time and increases thus linearly with time. In addition, it shows the Hamming distance to the root of the tree in orange. Saturation effects are visible as reduced Hamming distances that increase more slowly over time, but the effects are small. As expected, saturation effects are even less pronounced when comparing sequences to the root of the subtypes (here assumed to be in 1965, compare Fig. 1A) or a ‘founder’ sequence in 1980.

Figure 1C shows the analogous patterns for HIV-1. The Hamming distance of HIV-1 sequences from the inferred root of the HIV-1 group M tree (orange) is substantially lower than the RTT distance (blue) and increases only at about half the rate, suggesting substantial saturation. Similarly, the Hamming distances to the subtype consensus (only done for Subtypes B and C) increase less rapidly than the RTT distance, despite the fact that at 2–5 per cent sequence divergences from the subtype root saturation effects are unexpected. Such rapid saturation can arise through rate variation (Soubrier et al. 2012) or heavily skewed site-specific equilibrium frequencies resulting in rapid reversion (Halpern and Bruno, 1998; Hilton and Bloom 2018; Puller et al. 2020; Ho et al. 2005; Wertheim and Kosakovsky Pond 2011).

**Figure 2.**
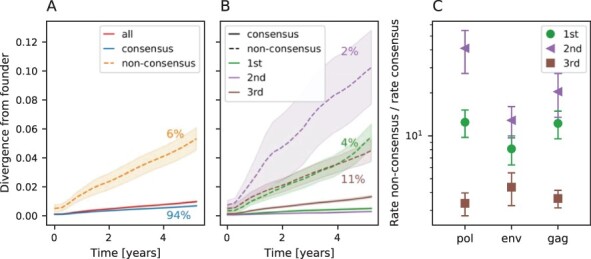
Divergence from founder sequence over time in the *pol* gene. (A) Divergence from founder overall and split for sites initially in consensus and non-consensus states. The reference used to define consensus and non-consensus sites is the HIV-1 group M consensus. Colored percentages are the fraction of sites corresponding to the related curve. Non-consensus sites represent only 6 per cent of the gene but diverge faster over time. Overall, 87 per cent of this divergence are due to reversions, while only 13 per cent are mutations toward another non-consensus nucleotide. (B) The data set from Panel A further split among the first, second, and third codon positions. The difference in evolution speed is greatest for nucleotides in the second position. (C) Ratio of non-consensus to consensus evolution rates computed from the curves in Panel B (Supplementary Figs. S3 and S4 for *env* and *gag*). The ratio is highest for second positions (triangles), where mutations can not be synonymous, followed by first and third positions.

We then performed a similar analysis on WH data on a 5-year timescale to determine whether similar rates and saturation effects exist within hosts. We compute WH evolutionary rates by measuring the divergence over time in Fig. 2D. Specifically, we calculate the divergence *d*(*t*) relative to a reference sequence, such as the root of the tree, according to:

(1) $$ d(t) = \frac{1}{N} \sum_{i}^{N} \delta_{i}^{ref}\cdot (1-f_{i}(t)) + (1-\delta_{i}^{ref}) \cdot f_{i}(t), $$

where *N* is the length of the region and $f_{i}(t)$ is the frequency of the founder nucleotide at position *i* and time *t* in the viral population. This founder nucleotide at each position *i* is approximated as the majority nucleotide at the first time point *t*_0_, and its frequency at each time point *t* is used to compute *d*(*t*). Details about the computation of the founder sequence can be found in Section 4.4. The Boolean $\delta_{i}^{ref}$ is such that $\delta_{i}^{ref} = 1$ if the founder nucleotide at position *i* is the same as in the reference sequence and $\delta_{i}^{ref} = 0$ otherwise. The first term $\delta_{i}^{ref}\cdot (1-f_{i}(t))$ in Equation (1) accounts for the change away from the founder at positions where the founder sequence equals the reference sequence. The term $(1-\delta_{i}^{ref}) \cdot f_{i}(t)$ accounts for the change at positions where the founder sequence differs from the reference sequence. In most cases, the founder nucleotide is replaced by the reference nucleotide and the population is getting more similar to the reference, and mutations to other states are ignored in this calculation (see below). When measuring the divergence relative to the founder sequence, Equation (1) simplifies to:

(2) $$ d(t) = 1 - \frac{1}{N} \sum_{i}^{N} f_{i}(t). $$

In all cases, the quantity *d*(*t*) measures the Hamming distance to the reference sequence expected for a randomly chosen sequence from the viral population of a sample. We then averaged the divergence trajectories of different patients and estimated uncertainty by bootstrapping groups of samples from the same patient with replacement. In analogy to the BH analysis, we use the root of the HIV-1 group M tree and subtype consensuses as reference sequences, supplemented by the founder sequence of each patient. Results are shown in Fig. 1D over a period of 5.5 years as the follow-up of most patients stopped after this duration. The filled areas represent one standard deviation of the bootstrap replicates. For more details about the methodology, see Sections 4.2 and 4.3.

Figure 1D shows that the divergence increases the fastest relative to the founder sequence at approximately $(16.1 \pm 1.1) \cdot 10^{-4}$ mutations per site per year. This rate is significantly and substantially higher than the rate at which RTT distance increases on the pandemic scale in line with previous observations that WH rate estimates tend to be higher (Alizon and Fraser 2013). Hamming distances to the subtype consensus or the root of the HIV-1 (M) tree increase significantly more slowly. In fact, these rates are compatible with their corresponding estimates at the pandemic scale (compare Panels C and D).

A ‘store-and-retrieve’ mechanism to explain the discrepancy between rate estimates should not only result in differences between BH and WH rate estimates (the rates at which the RTT distance and the distance to the founder sequence increase), but also for the rates at which Hamming distances to HIV-1 root or subtype consensuses increase. Since divergence to reference sequences decades in the past is increasing at compatible rates within and between hosts, these analyses suggest similar modes of divergence accumulation and do not support ‘store-and-retrieve’ as a primary mechanism to explain the discrepancy in rate estimates. In contrast, rate variation or rapid reversion is not expected to affect Hamming distance dynamics to fixed reference sequences like the HIV-1 (M) root. RTT distance estimates, however, are expected to be biased downward since rapid back-and-forth mutations are unaccounted for by the substitution models and do not contribute to the RTT distance. The observations in Fig. 1, and analogous results for *env* and *gag* regions shown in Supplementary Figs. S1 and S2, are thus compatible with saturation effects not captured by substitution models. We will now investigate WH dynamics of polymorphisms to show that rapid reversion to consensus states is a major contributor to this saturation.

### 2.2 Non-consensus sites diverge faster

Next, we explored the evolution toward and away from consensus within hosts in Fig. 2. Panel A shows the WH divergence separately at sites where the founder sequence agrees with the HIV-1 group M consensus and where it differs from it. Filled areas show the standard deviation of the bootstrap estimate. The divergence at sites where the founder sequence differs from the global consensus increases approximately seven times faster than in the rest of the sequence. A mutation at a site that initially differs from consensus could either be a reversion to consensus or a mutation to one of the two remaining nucleotides. We found that 87 and 85 per cent of mutations at these sites are reversion toward consensus for *pol* and *gag*, while this figure is 76 per cent for *env*. Mutations to a third state are thus a minor contribution. The sevenfold increased rate at 6 per cent of the sites that are initially non-consensus (in *pol*) implies that about one in three mutations bring the sequence closer to the HIV-1 root sequence (the number of reversion mutations divided by the total number of mutations: $\frac{7\cdot 0.06\cdot \mu}{(0.94 + 7\cdot0.06)\mu} \approx \frac{1}{3}$ where *µ* is the observed evolution rate.). This strong tendency to revert can explain the difference in evolutionary rates observed on WH and BH scales and is consistent with the threefold difference in slope between the divergence relative to the founder or HIV-1 group M root shown in Fig. 1D.

This accelerated evolution could be due to (1) reversion to an ancestral state to increase fitness or (2) reduced purifying selection at sites with high levels of diversity in global HIV-1 population. In order to differentiate between these possibilities, Fig. 2B shows the divergence by codon position. The degree to which divergence is accelerated differs among the first, second, and third positions in a codon. In particular, sites in the second position diverge the fastest when in a non-consensus state, while they diverge the slowest in a consensus state. This is consistent with the fact that second positions tend to be most conserved as only 2 per cent of such sites differ from the consensus sequence in *pol*.

Figure 2C quantifies the ratio of divergence rates at sites initially in a consensus or non-consensus state for *pol*, *env*, and *gag*. Details on the computation of these rates can be found in Section 4.3. In all cases, evolution rates of non-consensus sites are higher than consensus ones. The difference is greatest for second codon position sites, followed by first codon position sites (see Supplementary Figs. S3 and S4 for divergence plots for other genes). Mutations at second codon position sites are always non-synonymous and often cause drastic amino acid changes, while mutations at third codon position sites are often synonymous and generally less impactful. Mutations at first position sites can be both synonymous and non-synonymous. The observation that divergence is fastest at non-consensus but otherwise strongly conserved sites suggests that reversion mutations are selected to increase fitness and are not the result of reduced purifying selection at sites of high diversity. These results are consistent with previous observations showing that conserved sites tend to revert more quickly (Zanini et al. 2015) and the notion that selection for reversion is probably driven by the fitness costs of mutations that enabled immune escape in a previous host (Leslie et al. 2004). Such rapid reversion is an example of adaptation within hosts, but the combined escape-reversion dynamics on timescales spanning several transmission events looks like purifying selection at conserved sites.

### 2.3 Reversion mutations are positively selected

**Figure 3.**
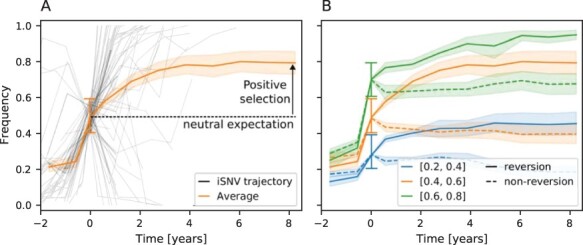
Positive selection on reversion mutations. (A) Frequency of reversion mutations seen between 0.4 and 0.6 frequency at one time point (offset to be *t* = 0) and their average over time. (B) Mean frequency over time for reversion (full lines) and non-reversion mutations (dashed lines) for different frequency windows (colors). Reversion trajectories are strongly selected for as their mean frequency increases over time. Non-reversion trajectories evolve close to the neutral expectation. The reference sequence used to define reversion mutations is the HIV-1 group M consensus. The solid orange line is the same in both panels.

If a lot of reversions are driven by selection, as the codon-position-specific analysis above suggests, effects of selection should be detectable in the dynamics of intra-host single nucleotide variants (iSNVs). Specifically, we expect to see a tendency of reversion mutations to increase in frequency and fix. We analyzed the frequency trajectories of iSNVs to look for such features. Similar to the previous analysis, we separate all trajectories into reversion and non-reversion groups and compare their evolutionary dynamics in Fig. 3. We select trajectories with at least one data point in a frequency interval $[f_{min}, f_{max}]$ for each group. We offset these trajectories in time so that *t* = 0 corresponds to their first data point seen in the frequency interval and compute the mean frequency of the trajectory group over time. The small minority of trajectories where both the initial nucleotide and the target nucleotide differ from the consensus sequences are classified as ‘non-reversions’ in this analysis. More details about the definition of trajectories and the methodology are in Supplementary Fig. S5 and Sections 4.5 and 4.6. We use the HIV-1 group M consensus sequence as a reference to define reversion mutations, but results are qualitatively similar when using subtype consensus or root sequence as a reference.

Figure 3A shows individual trajectories shifted to pass through the frequency interval [0.4, 0.6] at *t* = 0 along with their average. The mean frequencies for different initial conditions and groups of trajectories are shown in Fig. 3B. Since we condition the set of trajectories to start as minor variants and pass through a frequency interval at *t* = 0, we expect that trajectories tend to rise for *t* < 0, as is indeed observed. The dynamics at *t* > 0, i.e. after the time of conditioning, are informative about the selection of the iSNV. We do not expect any consistent trend to rise or fall in frequency for neutral mutations, hence their average frequency should be constant for *t* > 0. Contrary to that, we show in Fig. 3B that the frequency of reversion mutations increases on average over time. This suggests that these reversion mutations are beneficial on average and fix preferentially in the population, with probability given by the end point of the curves for each group of trajectories. This finding is consistent with the notion that the HIV-1 consensus sequence approximates a fitness optimum of HIV-1 (Zanini et al. 2017). On the other hand, non-reversion curves are flat or slightly decreasing for *t* > 0, suggesting that such mutations tend to be slightly selected against or are neutral—at least those that reach high frequency in the first place.

We note that the selection for reversion mutations is strongest for the *gag* region, see Fig. S6 for details. When splitting trajectories into synonymous and non-synonymous changes (irrespective of reversion/non-reversion), we observe that synonymous mutations tend to decrease in frequency for *t* > 0, while on average non-synonymous mutations increase, see Fig. S7. This suggests that high-frequency non-synonymous mutations tend to be beneficial, while synonymous mutations are slightly deleterious, consistent with earlier results (Zanini and Neher 2013). Common synonymous reversions, on the other hand, tend to further increase in frequency and fix preferentially, see Fig. S8.

### 2.4 Reversions can explain the rate mismatch

Over longer timescales, the rapid reversions we observe within hosts will lead to undetected substitutions along branches of the phylogeny whenever a mutation and its corresponding reversion happen on the same branch. When such reversion dynamics are not captured by the substitution models, the evolutionary rate inferred by phylogenetic methods will be too low (Halpern and Bruno 1998; Hilton and Bloom 2018; Puller et al. 2020). Here we explore how much of the discrepancy between evolutionary rates estimates at the WH and BH scales can be attributed to rapid reversions not being properly captured by substitution models.

We quantify the impact of reversions on the BH evolution rate using an evolutionary model that accounts for the reversion bias we observed within hosts. We use the TreeTime library (Sagulenko et al. 2018) to define a site-specific general time-reversible (GTR) model (Puller et al. 2020). We parameterize the mutation rate from nucleotide *j* to *i* at position *α* as:

(3) $$ Q_{ij}^\alpha = \mu p_i^\alpha W_{ij}, $$

where *µ* is the mean mutation rate per site per year, $p_i^\alpha$ describes the equilibrium probability of finding nucleotide *i* at site *α*, and *W*_*ij*_ accounts for the overall variation in rate between different nucleotide pairs *i* and *j* independent of position (i.e. the differences between transitions and transversions). We use $\mu = 16.1\cdot 10^{-4}$, the overall WH evolution rate observed in Fig. 1D. In this model, the bias for reversion is introduced via the equilibrium frequencies $p_i^\alpha$. These depend on the genome position *α*, enabling us to skew the frequencies toward the consensus nucleotide at each position. Contrary to common evolutionary models that include rate variation between sites, we keep the evolutionary rate constant across positions and vary $p_i^\alpha$ instead. However, our results show little change if a gamma-distributed rate variation is incorporated, especially when the shape parameter is greater than 2. We choose $p_i^\alpha$ such that the model reproduces the WH rates of reversions and evolution away from consensus. Specifically, we use

(4) $$ \begin{matrix} p_i^\alpha &amp; = \frac{\mu_-^\alpha}{\mu_-^\alpha + \mu_+^\alpha} &amp; \textrm{if} \qquad i = \textrm{consensus at } \alpha \end{matrix}, $$

(5) $$ \begin{matrix} p_i^\alpha &amp; = \frac{\mu_+^\alpha}{\mu_-^\alpha + \mu_+^\alpha}\cdot r_i^\alpha &amp; \textrm{if} \qquad i \neq \textrm{consensus at } \alpha \end{matrix}, $$

where $\mu_+^\alpha$ and $\mu_-^\alpha$ are the consensus and non-consensus divergence rates, respectively, computed from WH data shown in Fig. 2B. These rates reproduce the equilibrium frequencies in a model with two states (consensus and non-consensus). These rates are codon position-specific, meaning for every *α* that is a first codon position $\mu_+^\alpha = \mu_+^{1st}$ and $\mu_-^\alpha = \mu_-^{1st}$ and analogously for the second and third codon positions. The parameter $r_i^\alpha$ is used to specify the relative proportions of the three non-consensus nucleotides. It is chosen so that 85 per cent of the non-consensus nucleotides are the transitions from the consensus, while the two transversions contribute 7.5 per cent each. These values were inferred from the BH alignment and are consistent with the WH observations, see Section 2.2. Otherwise, this GTR model is purely informed by WH rates.

We then used this model to simulate evolution along an HIV-1 phylogeny and generate a multiple sequence alignment (MSA) using TreeTime and the inferred HIV-1 root sequence (as used in Fig. 1). We then inferred a tree from the MSA generated using IQ-TREE, as we did for the real data.

**Figure 4.**
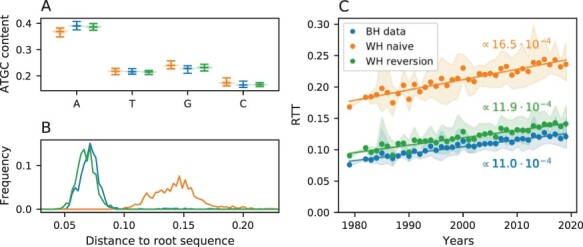
Substitution models that account for reversions can largely explain the rate mismatch. This figure shows the sequence diversity and RTT distances for simulated data generated with a substitution model that accounts for reversion, parameterized by WH data (WH reversion) and a model that does not account for reversion (WH naive) for the *pol* region. (A) Violin plot of the nucleotide content for the BH data and the MSAs generated. The naive model tends to equilibrate the nucleotide composition. (B) Histogram of Hamming distances to the root sequence. The reversion-informed model agrees well with the BH observations, while the naive one generates sequences very far from the root. (C) While the RTT distance estimated from data generated by the naive model is consistent with the evolution rate we used in the model (true value $16.1 \cdot 10^{-4}$ per site and year), the data generated using the model with reversion results in much lower estimates, similar to the rate estimated from BH data. The results for *env* and *gag* are shown in Supplementary Figs. S9 and S10.

Figure 4 compares the diversity of original and generated MSAs and the length of the inferred trees to quantify the impact of reversions on phylogenetic inference. A model that does not account for reversions, i.e. where $p_i=0.25$ for $i \in \textrm{A,C,G,T}$ for all sites, was included for comparison and is referred to as the naive GTR model. Figs. 4A and 4B show a comparison of the real and generated MSA characteristics. The MSA generated using our WH-informed GTR model (green) has a similar nucleotide content and distance to the root as the real BH data (blue). On the contrary, the naive GTR model that does not take reversions into account (orange) results in a more diverse MSA and overall nucleotide content that is less similar to the BH data.

Figure 4C shows that the evolutionary rate estimated from the RTT regression of the tree reconstructed from the MSA simulated using the naive GTR model is, as expected, very close to the WH evolution rate of $\mu = 16.1\cdot 10^{-4}$ mutation per site per year we input into the model. Our custom GTR model, which uses the same *µ* but accounts for reversions, results in a RTT regression with a slope of $11.9\cdot 10^{-4}$, substantially lower than the true rate and within 10 per cent of the rate estimate from the RTT regression for the original phylogenetic tree. This suggests that a substitution model parameterized by rates and reversion propensity of WH evolution can largely reconcile the discrepancy of rate estimates at different scales, even if it does not include rate variation between different sites.

We find qualitatively similar results for the *gag* (see Supplementary Fig. S10). In the case of *env*, the tree reconstructed from the data generated and subsequent analysis is unreliable due to excessive saturation in the model (see Supplementary Fig. S9).

## 3. Discussion

Evolutionary rate estimates depend strongly on the timescale over which they are measured (Ho et al. 2005; Aiewsakun and Katzourakis 2016). Here, we explored this effect on the scale of the HIV-1 pandemic, individual subtypes, and within hosts. We showed how observations on short timescales give rise to patterns on longer scales. Differences between rate estimates WH scale and on the pandemic scale can, to a substantial degree, be explained by a strong tendency to revert deleterious mutations to their preferred state. These unpreferred states are probably the result of escape from immune selection in a previous host, which gradually revert as the host-specific selection pressure is relaxed in future hosts. Microscopically, we thus observe evolutionary dynamics driven by the adaptation to a changing environment: both changes, escape and reversion, are beneficial in their respective environments. These transiently beneficial escape mutations are generally deleterious on longer timescales, such that the aggregate effect of this dynamic looks like slowly acting purifying selection (Wertheim and Kosakovsky Pond 2011).

Substitution models commonly used to reconstruct phylogenies and infer evolutionary rates do not account for rapid reversions, which would require site-specific preferences for different states (Halpern and Bruno 1998; Hilton and Bloom 2018; Puller et al. 2020). We explored the effect of reversions on phylogenetic inference and rate estimates of HIV-1 by defining a simple site-specific model parameterized by reversion and non-reversion rate estimates from longitudinal data within hosts. Phylogenetic inference from data simulated using this model revealed that reversions during chronic infection can explain approximately 90 per cent of the apparent slowdown of evolution for the *pol* gene of HIV-1. A similar selection for reversion mutations has also been observed during acute infection (Boutwell et al. 2010; Leslie et al. 2004) or the transmission bottleneck (Carlson et al. 2014). Such preferential transmission of consensus-like variants could amplify the overall effect of incomplete reversions during chronic infection. Together, these results suggest that, among the hypotheses proposed to explain the difference in rates (Lythgoe and Fraser 2012; Redd et al. 2012; Zanini et al. 2015; Leslie et al. 2004), ‘adapt and revert’ is the main mechanism.

The strongest effects of unaccounted reversions in phylogenetic inference are expected on long branches in the phylogenetic tree, where mutations are masked by their corresponding reversions (Hilton and Bloom 2018; Puller et al. 2020). The well-known phenomenon of long-branch attraction can, in these cases, already set in for branches that are nominally quite short. A strong tendency to revert can lead to sites that are completely saturated, yet almost always are in the same state—an effect not captured well by rate variation.

Rapid reversions are probably essential to conserve global fitness by purging costly immune escape mutations acquired in individuals earlier in the transmission chain (Carlson et al. 2014; Zanini et al. 2017). In addition to reversion, fitness costs of escape mutations can, of course, also be mitigated by compensatory mutations (Crawford et al. 2007; Carlson and Brumme 2008). Although such compensatory mutations presumably slow down many reversions, we still observe a marked difference in iSNV frequency dynamics toward vs. away from consensus. In addition, compensatory evolution can change the preferred sequence to a new local fitness maximum to which mutations revert, adding an additional slow timescale to the evolutionary process. We expect the preferred sequence to slowly drift on timescales much longer than the typical serial interval along transmission chains. This effect has been observed in deep mutational scanning experiments in influenza viruses (Hilton and Bloom 2018; Doud et al. 2015). Such effects are also consistent with the ‘Prisoner of War’ model by Ghafari *et al.* (2021), where a slowly changing fitness landscape (through host switches, host adaptation, or compensatory evolution) gives rise to apparent rates of evolution that decrease with the timescale of observation over many orders of magnitude.

The star-like diversification of HIV-1 into multiple subtypes gives a clear notion of a consensus sequence that can be used to approximate a putative fitness peak toward which reversions occur. In other viruses, for example, influenza A viruses, the ladder-like or otherwise structured phylogenies do not allow a straightforward definition of a consensus sequence. Nevertheless, it is possible that adaptation to a changing immunity landscape and reversions contribute with a similar magnitude to sequence turnover.

## 4. Materials and Methods

### 4.1 Data set and filtering steps

#### 4.1.1 Between-host data sets

Our BH data sets come from the LANL HIV databases. All HIV-1 group M sequences with exact sampling date were downloaded for the *pol*, *env*, and *gag* regions. Subtype O and N sequences were filtered out. Sequences with ambiguous nucleotides and sequences labeled as ‘problematic’ on LANL website were removed. Only one sequence was kept per patient. The data sets were downloaded on 14 July 2021. This gave us a total of 6,649 sequences for *pol*, 15,034 for *env*, and 8,948 for *gag*.

Regarding each genomic region, we subsampled the data set to have 1,000 sequences in each case, with the same number of sequences for each year where sequences were available (except for early years where fewer sequences were available). For each region, Subtype B represents approximately 40 per cent of all sequences, Subtype C approximately 15 per cent, and the rest encompass the other subtypes or unlabeled subtypes. Subtype B sequences are more common in early years while Subtype C sequences represent a larger proportion in recent years. We then performed an MSA, including the reference HIV-1 HXB2 sequence, using Multiple Alignment using Fast Fourier Transform (Katoh and Standley 2013) and the Nextstrain framework (Huddleston et al. 2021). Insertions relative to the reference HXB2 sequence were removed. We removed all positions of the alignment where more than $10\%$ of sequences have a gap as the alignment can be unreliable in such positions. The alignment for the *pol*, *env*, and *gag* regions are the data sets used for our BH analyses. See the section Code and data availability for access to the data sets.

#### 4.1.2 Within-host data sets

Our WH analysis leverages the time resolution of the HIVEVO data set (Zanini et al. 2015). This data set is freely available with tools made available to facilitate the analysis. We use these tools to obtain a three-dimensional matrix of nucleotide frequencies for each patient. The three axes of these tables are the HIV-1 genome position, the nucleotide, and the time since infection of the sample. Each entry in these matrices gives the frequency of a given nucleotide at a given position on the genome at this time point, relative to the total intra-patient HIV-1 population. These matrices form our WH data set. We excluded patients *p7* and *p10* from our analysis as their samples were very uneven in time or because there was evidence of multiple founder sequences.

The estimates of nucleotide frequencies are unbiased in the $[0.1, 0.9]$ range, while coverage and depth are globally sufficient (Zanini et al., 2016). We applied several filtering steps prior to analysis to avoid biases in our results. We masked data points with sequencing coverage inferior to 100 and/or where the depth was low. We also removed genome positions that were not mapped to the consensus sequence and/or seen to be too often gapped in the MSA of BH sequences. The alignment and mapping of such sites can be unreliable; thus, we removed them from our analysis. This filtering procedure is mainly relevant for the *env* gene, which is the region with the most noise.

### 4.2 Distance and divergence over time

The first result section gives an overview of the method used to compute the distance and divergence over time in Fig. 1 and Supplementary Fig. S1 and S2. Additional details are given below.

Hamming distances were computed by counting the number of sites that do not match the reference sequence for each sequence in the data set. We then divide this number by the length of the sequence to obtain the relative distance to the reference. Hamming distances were computed using three reference sequences. The first is the root sequence of the tree. The tree was inferred using the IQ-TREE GTR+F+R10 model (Minh et al. 2020), while the root sequence was computed using TreeTime ancestral reconstruction on this tree (Sagulenko et al. 2018). We chose to use the root sequence instead of the consensus sequence of the alignment in Figs. 1 and 4 to avoid biases due to over-representation of Subgroup B and C sequences. The second and third reference sequences are Subgroup B and C consensus sequences. See Section 4.4 for details on the computation of consensus and founder sequences. To compute the Hamming distances to the subtype consensus, we averaged the distances computed for Subtype B and C sequences relative to their consensus. The average was then weighted by the relative number of each subtype sequence in each year.

The RTT distances shown in Figs. 1 and 4 and Supplementary Figs. S1, S2, S9, and S10 are computed directly from the tree generated via IQ-TREE. Such distances were computed for every leaf of the tree (i.e. every sequence in our data set) and then averaged for sequences sampled in the same year for visualization. Taking into account the phylogenetic information allows the detection of some mutations that occur and then revert along the tree. Consequently, the estimates of the RTT distance are higher than the Hamming distance ones.

### 4.3 Evolutionary rates

The evolutionary rates in Figs. 1 and 2C and Supplementary Figs. S1 and S2 are the slopes of linear fits of the data. For the BH plots (Fig. 1C and Supplementary Figs. S1A and S2A), the fit was done on the data from 1979 to 2022. For the WH plots (Figs. 1D and 2C and Supplementary Figs. S1B and S2B), we estimated a linear fit from 200 to 2,000 days in the infection. We removed the first 200 days from the fit as for most patients the first sample we have is in the 0–200 days window. This causes the small flat part of the founder curves near *t* = 0, which could bias our evolution rate estimates. Consequently, we decided to only use data starting from 200 days into the infection for the fit, which is more than enough to get an accurate estimate of the slope. For the WH rate estimates, we estimate the error by bootstrapping patients.

Estimating confidence intervals for evolutionary rates at the level of the pandemic is challenging because of the phylogenetic relationship and shared ancestry of the sequences. Instead of using probabilistic phylogenetic models, which suffer from residual recombination and model inadequacies, we opted for phylogenetic boot-strapping procedure for the BH rate estimates. Specifically, we cut all branches of the time-scaled phylogenetic trees at the year 1980 and thereby obtain a collection of subtrees. Sequences in the same subtree are correlated, but they are not correlated with sequences on another subtree (as evolution happens on different branches of the original tree). We performed bootstrapping to estimate distances and rates by sampling with replacement from sequences in these subclades. The errors provided for the rate estimates in Fig. 1 and Supplementary Figs. S1 and S2 are computed from these bootstrap estimates. The HIV-1 pandemic has undergone a large radiation in 1960s and 1970s, which makes such bootstrap estimates possible.

### 4.4 Consensus and founder sequence

Consensus sequences were computed from our BH data sets. We computed three consensus sequences for each region studied. The first is the HIV-1 group M global consensus, which is the majority nucleotide of the alignment at each position. The second and third are the Subtype B and C consensus sequences. These were computed in the same way, using a subset of the alignment that contains only the sequences of the subtype in question.

The founder sequence is an approximation of the sequence of the virus at the time of infection in a patient. They are computed from our WH data set for each patient separately. The founder sequence is the majority nucleotide in each position from the first sample of each patient. In this sense, it is the consensus sequence obtained from the first sample of each patient. For most patients in our data set, the first sample is taken at approximately 90 days after infection and no data are available on the early phase of infection. Consequently, the founder sequence computed is an approximation of the original virus.

### 4.5 Trajectory extraction and metadata

A trajectory is a sequence of nucleotide frequencies and associated time. Each trajectory corresponds to one genome position and one nucleotide only. We extracted trajectories from our WH data set according to several criteria. Firstly, every trajectory must be extinct before the first point, i.e. we consider only new mutations. This is to avoid biases that could be due to immune interaction existing already. Secondly, frequencies must be between 0.01 and 0.99 at all time points. The trajectory is considered extinct if it is below 0.01 and fixed if above 0.99. Lastly, we apply a mask to data points according to what is shown in Section 4.1. Trajectories that have their first and/or last points masked are removed from the analysis.

Every trajectory extracted according to the criteria above is coupled with its metadata. This contains all the relevant information, such as whether the mutation is a reversion or not and whether it fixed or was lost. This information is used to create subgroups of trajectories. From these subgroups, one can study the impact of a trait associated with a mutation for WH evolution, as shown in Fig. 3 and Supplementary Fig. S5 for reversion and non-reversion trajectories.

### 4.6 Mean frequency in time

While looking at divergence values informs us about the global evolution of the WH population, it cannot tell us whether the mutations we see on non-consensus sites are actually reversions to the consensus state or simply mutations to another nucleotide. This motivated us to look directly at the evolution of new mutations independently by observing their frequency trajectories in time. Trajectories were extracted and filtered according to Sections 4.1 and 4.5. Despite these filtering steps, our data are inherently biased toward small and/or low-frequency trajectories which are more common. In order to alleviate this bias, we compare reversion and non-reversion trajectories in the same manner. Accordingly, the resulting signal can be attributed to the effect of being a reversion (or not).

Due to the limited number of trajectories available and the often lack of information about trajectory fixation, for example, because it is still active at the last sample, the probability of fixation plots were not adequate for our analysis. We, thus, decided to pay attention to the evolution of the mean frequency in time for groups of trajectories. Trajectories were grouped in frequency bins, as described in the main text, to avoid bias toward positively or negatively selected trajectories. Supplementary Fig. S5 illustrates how this was done. Sometimes a trajectory’s first pass through the frequency window is missed and only caught on the second pass, which results in a few trajectories that enter the frequency window from above. This happens when the frequency of a mutation changes drastically from one sample to the next, i.e. the reported frequency jumps directly from below to above the window. Nevertheless, these are ‘new’ mutations as they were not seen in the first sample of the patient. We kept these trajectories to avoid potential bias, but including or excluding them does not have a big impact on the final results.

We then created time bins of 400 days from 600 days before up to 3,000 days after a trajectory is seen in a frequency window. We compute the average frequency of all trajectories belonging to the same group in each time bin. A trajectory contributes its current frequency if a data point is available at this time and does not contribute if no data are available in that time bin. Trajectories that fixed in the population contribute with a frequency of *f* = 1 to time bins subsequent to their fixation. Similarly, lost trajectories contribute *f* = 0 to time bins subsequent to their disappearance in the viral population. Trajectories that are still active after their last data point (because the study stopped before it could fix or be lost) contribute the frequency of their last data point to the following time bins.

## Supplementary Material

veac118_Supp

## Data Availability

The code and data used for the analysis can be found at https://github.com/neherlab/HIVEVO_reversion. Due to issues with the data sets’ size, only intermediate BH and WH data files in a compressed format are found in the github folder. A link to the full data set is available there. Scripts are present to reproduce the results shown in this paper.
