# Supplementary material for: Reversions to consensus are positively selected in HIV-1 and bias substitution rate estimates
Source: Virus Evol. 2022 Dec 15;9(1):veac118. doi: 10.1093/ve/veac118 (PMC9829961; doi:10.1093/ve/veac118)
Supplement: veac118_Supp [file veac118_supp.zip › supplement.pdf]

Supplementary materials: Reversions to consensus are positively selected in HIV-1 and bias substitution rate estimates

Valentin Druelle[1,2] and Richard A. Neher[1,2]

[1]Biozentrum, University of Basel, Basel, Switzerland
[2]Swiss Institute of Bioinformatics, Basel, Switzerland

December 7, 2022

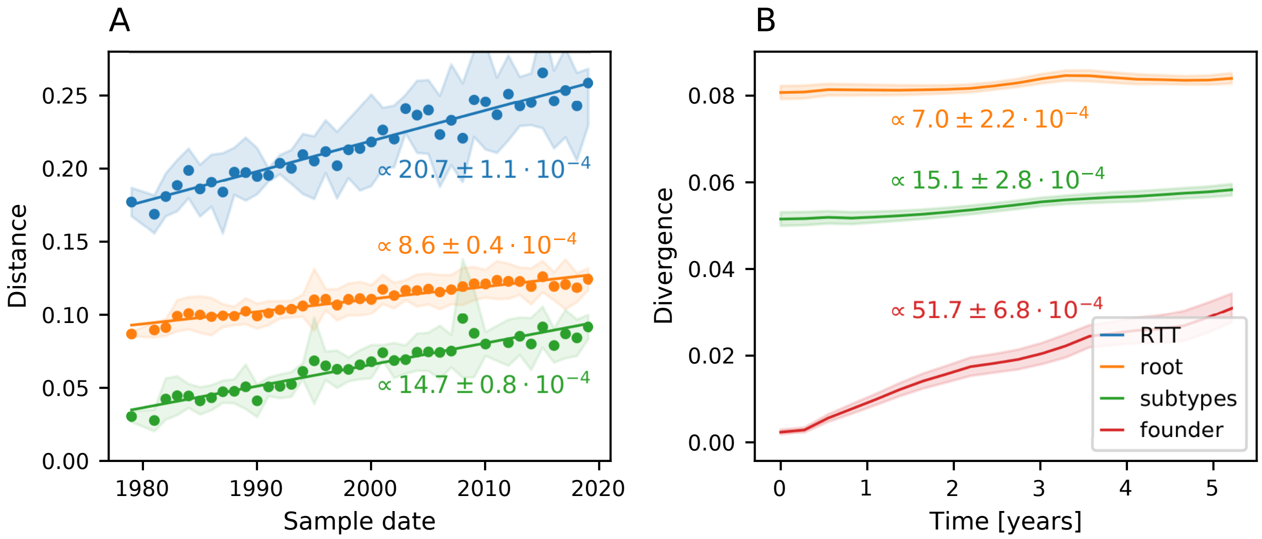

Supp. Fig. S1: Corresponds to Figure 1, for the *env* gene. The Y axes of panel A and B are not shared in this case as the RTT distance is much higher than what we observe within host. The relative difference between the rates is higher than what is seen for the *pol* and *gag* genes. This is consistent with the fact that *env* mutates faster overall, which would also lead to more reversions.

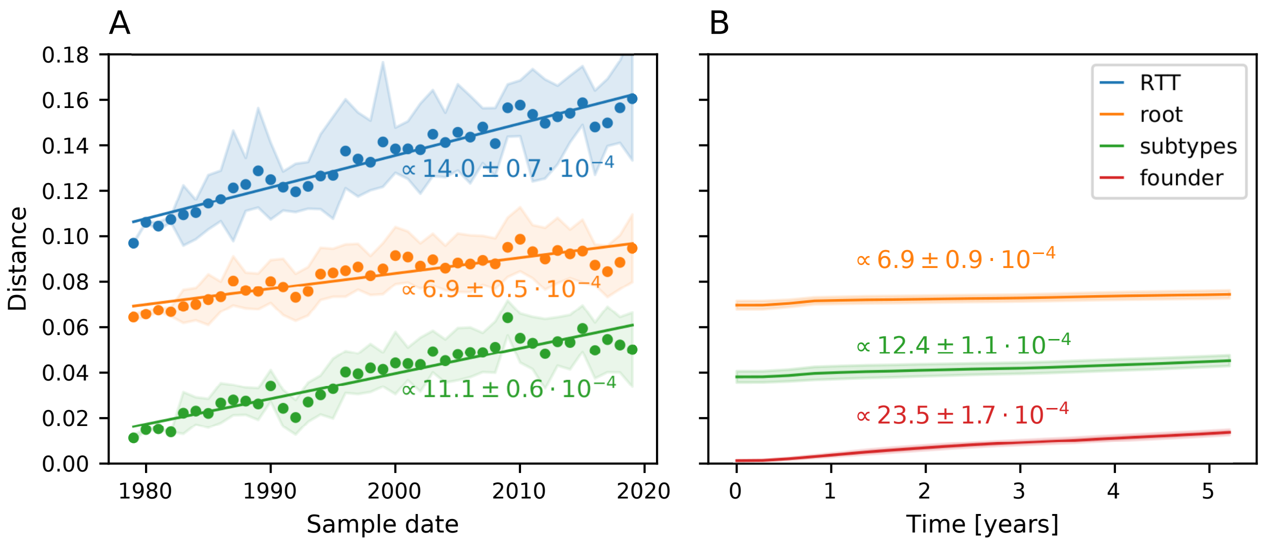

Supp. Fig. S2: Corresponds to Figure 1, for the *gag* gene. The overall mutation rate is slightly higher than for the *pol* gene but the relative difference between the rates is similar.

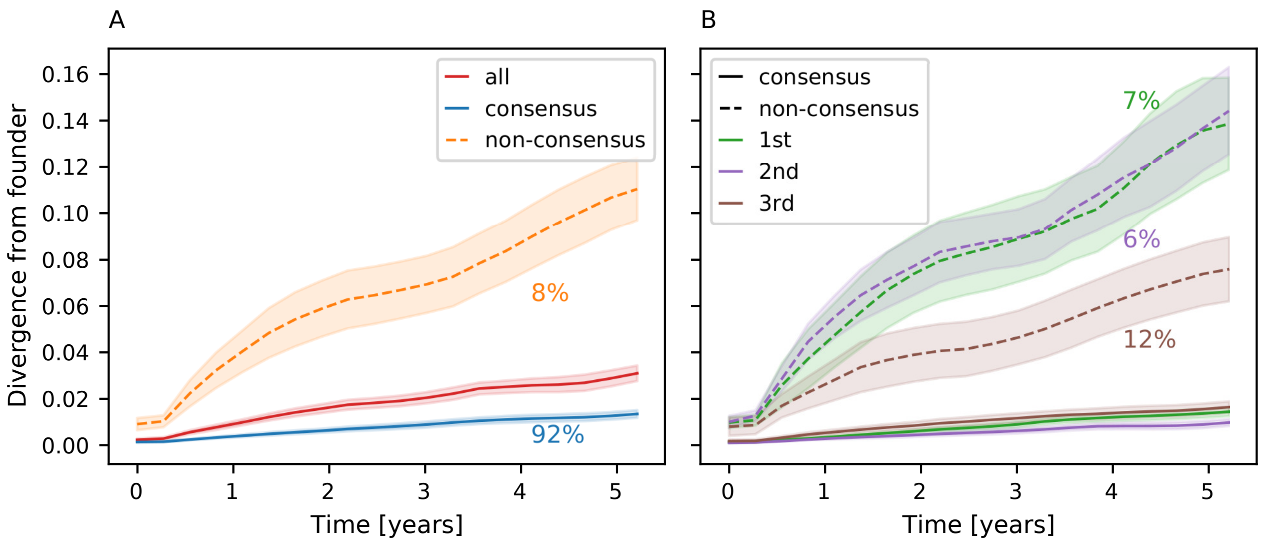

Supp. Fig. S3: Corresponds to Figure 2, for the *env* gene. In this gene, non-consensus sites at the 1st and 2nd codon position seem to diverge at a similar rate, suggesting a comparable selection for such mutations. 3rd codon position sites in a non-consensus state still diverge the slowest.

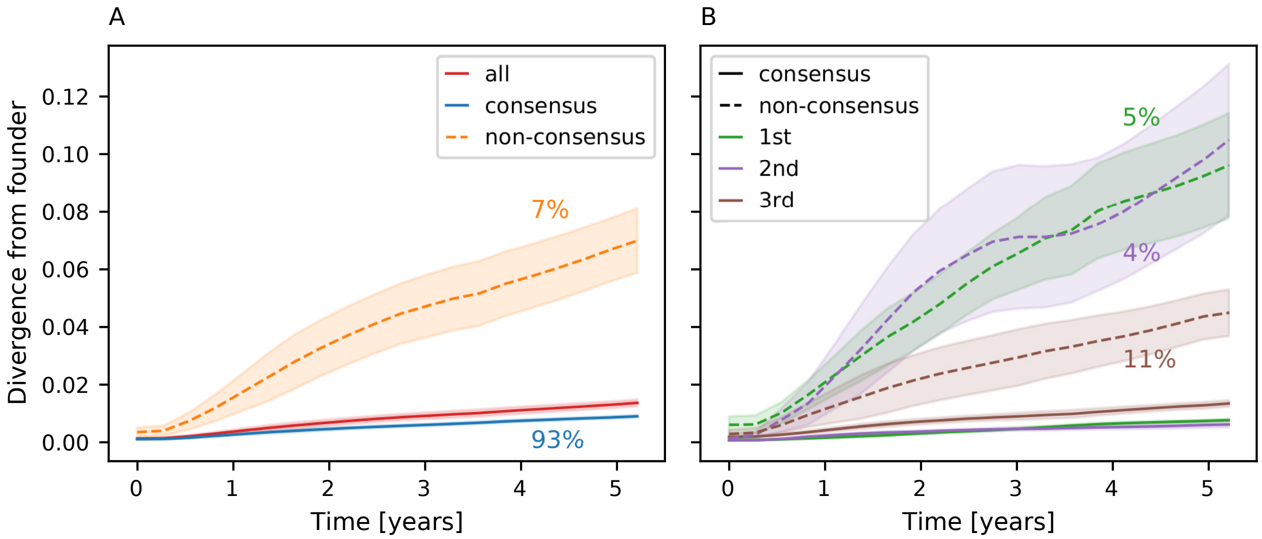

Supp. Fig. S4: Corresponds to Figure 2, for the *gag* gene. Similar to the *region*, non-consensus sites at the 1st and 2nd codon position diverge at similar rates. Non-consensus sites in the 3rd codon position still diverge the slowest, consistent with the fact that mutations at such sites are often synonymous and consequently under less selection pressure.

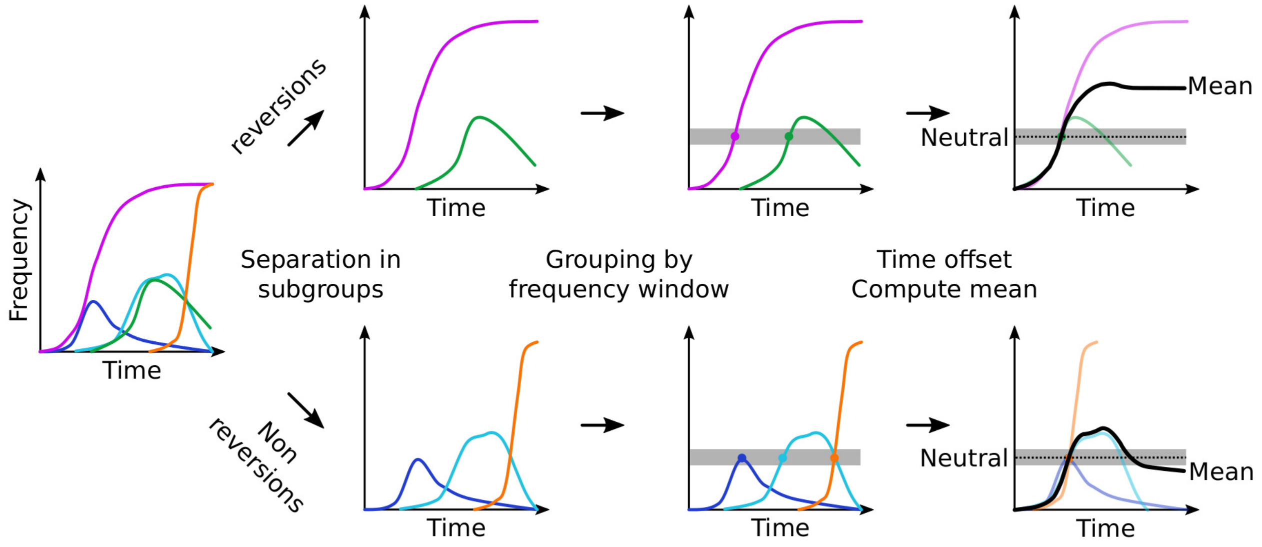

Supp. Fig. S5: Sketch of the methodology used to compute the curves shown in Figure 3 as described in the main text and section M&M 1.2. Trajectories are divided into reversion and non-reversion mutations. From each of these subgroups, trajectories that have one data point in the given frequency window are grouped together and offset in time so that this data point corresponds to t=0. We compute the mean of these trajectories and plot it in Figure 3.

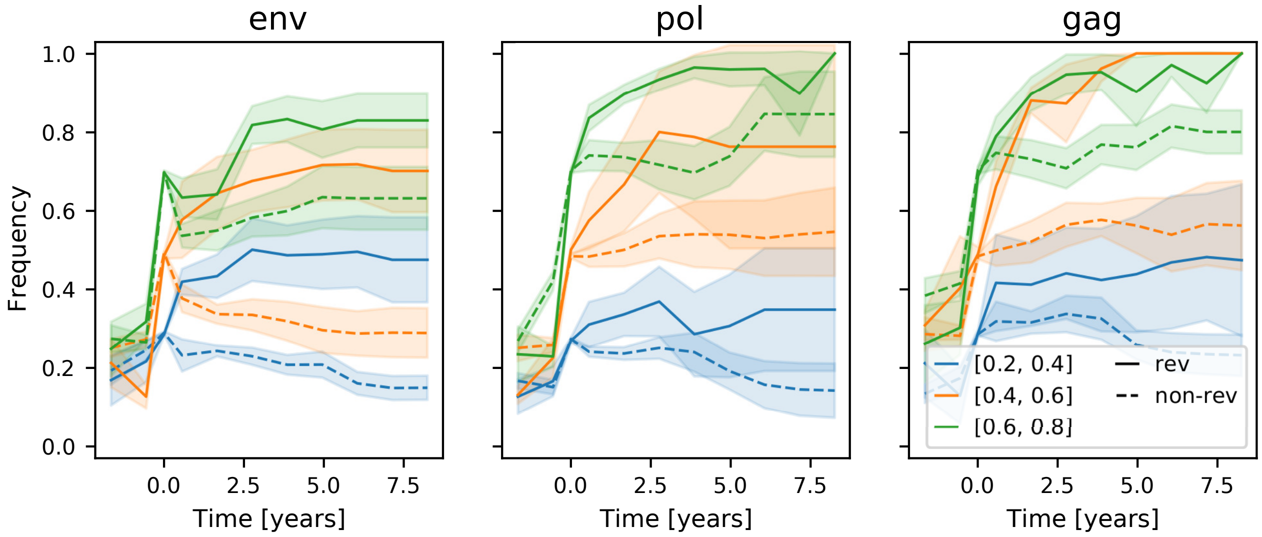

Supp. Fig. S 6: Corresponds to Figure 3B, split by gene. Selection for reversion is strongest in the *gag* region and weakest in the *env* region.

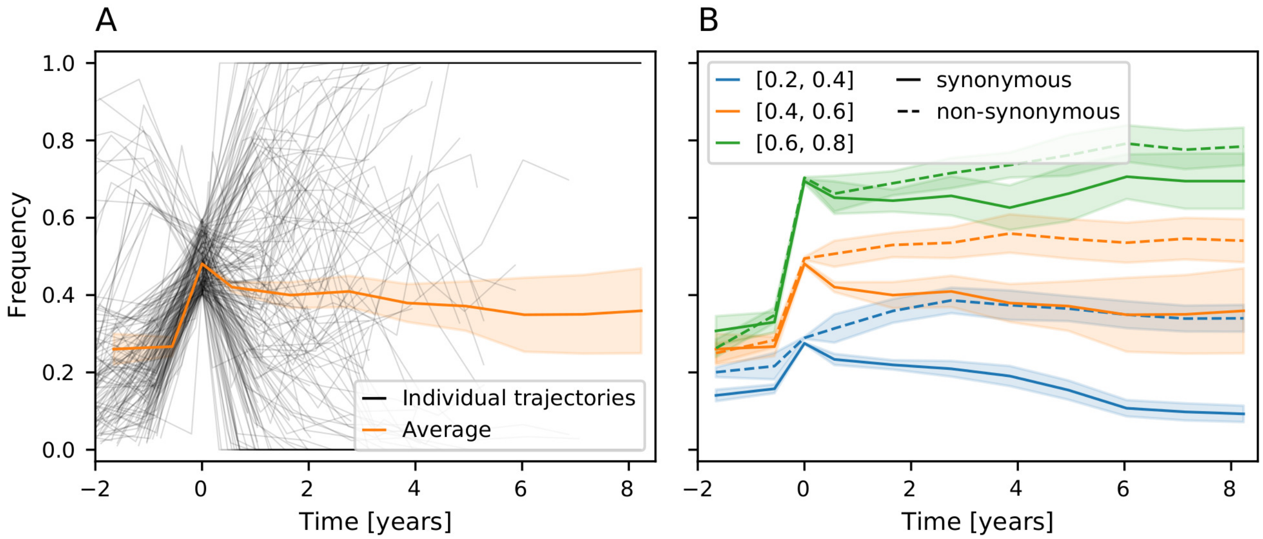

Supp. Fig. S 7: Corresponds to Figure 3, for synonymous and non-synonymous trajectories. Overall synonymous mutations are selected against and non-synonymous mutations seem to be selected for, but the effect is smaller than what we see for reversions and non-reversions in Figure 3.

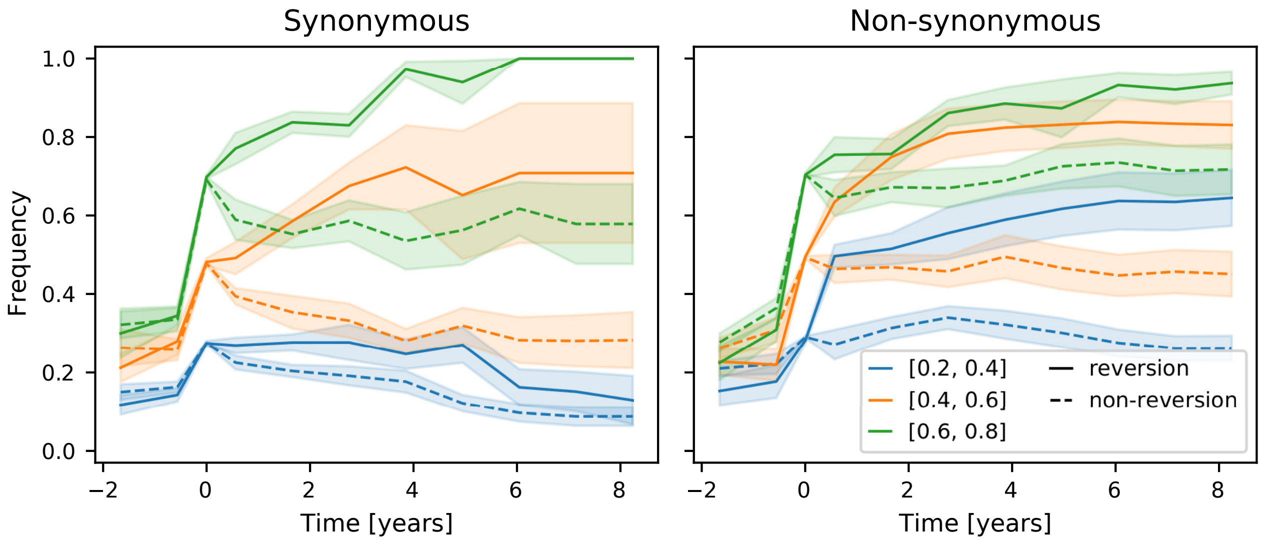

Supp. Fig. S8: Corresponds to Figure 3B, with a split between synonymous trajectories only (left) and non-synonymous trajectories only (right). Overall reversion mutations are selected. Interestingly, synonymous reversions seem to be selected more strongly than non-synonymous ones at higher frequencies, but the opposite is true at lower frequencies.

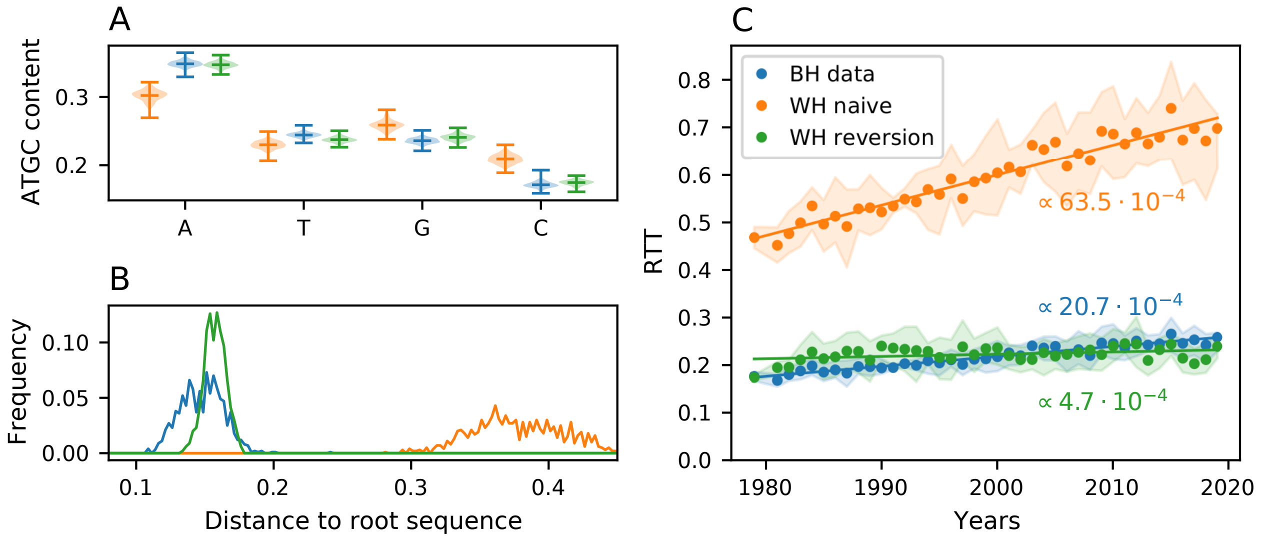

Supp. Fig. S9: Corresponds to Figure 4, simulated for the *env* gene. The WH mutation rate in this region is so high that the reversion model attenuates most of the clock signal. This leads the tree reconstruction to fail and underestimates the evolution rate for the WH reversion model in this case.

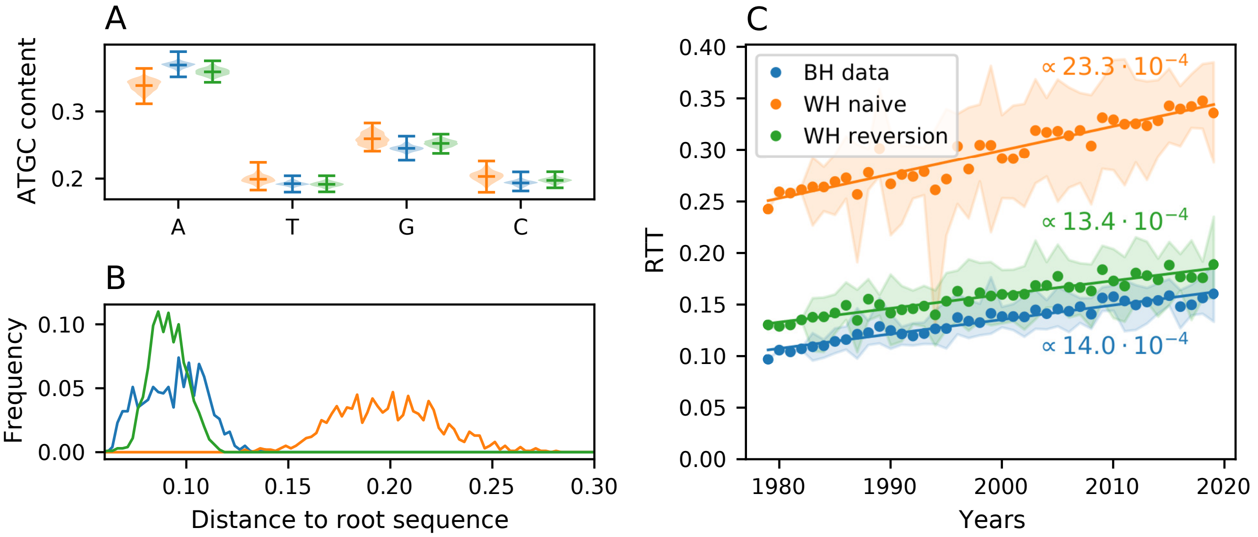

Supp. Fig. S10: Corresponds to Figure 4, simulated for the *gag* gene. The WH reversion model matches the between host observations better in this region as well, with relative differences in observed evolution rates that are similar to the *pol* region.
